# Episodic memory deficit in HIV infection: common phenotype with Parkinson’s disease, different neural substrates

**DOI:** 10.1007/s00429-023-02626-x

**Published:** 2023-04-18

**Authors:** Rosemary Fama, Eva M. Müller-Oehring, Taylor F. Levine, Edith V. Sullivan, Stephanie A. Sassoon, Priya Asok, Helen M. Brontë-Stewart, Kathleen L. Poston, Kilian M. Pohl, Adolf Pfefferbaum, Tilman Schulte

**Affiliations:** 1 Department of Psychiatry and Behavioral Sciences, Stanford University School of Medicine, 401 Quarry Rd, Stanford, CA 94305 USA; 2 Neuroscience Program, Center for Health Sciences, Bioscience Division, SRI International, 333 Ravenswood Ave, Menlo Park, CA 94025 USA; 3 Department of Neurology and Neurological Sciences, Stanford University School of Medicine, Stanford, CA 94305 USA; 4 Department of Neurosurgery, Stanford University School of Medicine, Stanford, CA 94305 USA; 5 Clinical Psychology, Palo Alto University, 1791 Arastradero Rd, Palo Alto, CA 94304 USA

**Keywords:** HIV infection, Episodic memory processes, Regional brain volume, Parkinson’s disease

## Abstract

Episodic memory deficits occur in people living with HIV (PLWH) and individuals with Parkinson’s disease (PD). Given known effects of HIV and PD on frontolimbic systems, episodic memory deficits are often attributed to executive dysfunction. Although executive dysfunction, evidenced as retrieval deficits, is relevant to mnemonic deficits, learning deficits may also contribute. Here, the California Verbal Learning Test-II, administered to 42 PLWH, 41 PD participants, and 37 controls, assessed learning and retrieval using measures of free recall, cued recall, and recognition. Executive function was assessed with a composite score comprising Stroop Color-Word Reading and Backward Digit Spans. Neurostructural correlates were examined with MRI of frontal (precentral, superior, orbital, middle, inferior, supplemental motor, medial) and limbic (hippocampus, thalamus) volumes. HIV and PD groups were impaired relative to controls on learning and free and cued recall trials but did not differ on recognition or retention of learned material. In no case did executive functioning solely account for the observed mnemonic deficits or brain-performance relations. Critically, the shared learning and retrieval deficits in HIV and PD were related to different substrates of frontolimbic mnemonic neurocircuitry. Specifically, diminished learning and poorer free and cued recall were related to smaller orbitofrontal volume in PLWH but not PD, whereas diminished learning in PD but not PLWH was related to smaller frontal superior volume. In PD, poorer recognition correlated with smaller thalamic volume and poorer retention to hippocampal volume. Although memory deficits were similar, the neural correlates in HIV and PD suggest different pathogenic mechanisms.

**Supplementary Information:**

The online version contains supplementary material available at 10.1007/s00429-023-02626-x.

## Introduction

Even in the current era of antiretroviral therapies (ART), mild to moderate cognitive deficits can be observed in people living with HIV infection (PLWH) (Heaton et al. 2010). As PLWH live longer, the most susceptible cognitive deficits to the progression of impairment in aging include episodic memory and motor functions, which may be associated with hippocampal (Maki et al. 2009) and basal ganglia (Goodkin et al. Sep 2017) dysfunction. The behavioral- and brain-related impairments observed in HIV infection are more often consistent with age-related diseases that affect subcortical regions such as Parkinson’s disease (PD) than age-related diseases that affect cortical regions, such as Alzheimer’s disease (Scott et al. Aug 2011), although the historical and heuristic dissociation between “subcortical” and “cortical” dementia has continued to diminish. For example, hippocampal dysfunction has been reported in HIV (Maki et al. 2009), and hippocampal atrophy, characteristic of cortical dementias, has been observed in PD (Nagano-Saito et al. 2005; La 2019; Uribe et al. 2018).

PLWH often exhibit episodic memory deficits (Fama et al. 2009, 2016; Doyle et al. 2019; Tan et al. 2013; Woods et al. 2009). In the pre-ART era, episodic memory deficits in HIV were attributed to executive dysfunction, component cognitive processes related to frontostriatal dysfunction including difficulty producing and using strategies to learn or recall information, slowed speed of information processing, rather than to a mnemonic impairment. Although this was also the thinking behind episodic memory deficits observed in other neurological diseases with cognitive deficits associated with frontostriatal dysfunction (i.e., PD) (Massman et al. 1990), there has been increasing support of concomitant encoding (Chiaravalloti et al. 2014; Siquier and Andres 2021; Weintraub et al. 2004) and consolidation deficits (Chiaravalloti et al. 2014; Bronnick et al. 2011). Taken together these results support the relevance of encoding, consolidation, and retrieval processes to episodic memory performance; however, these component mnemonic processes are difficult to dissociate. Recently, a study examined verbal learning and memory in PLWH, individuals with Huntington’s disease (HD, primarily affecting subcortical structures), and individuals with temporal lobe epilepsy with mesial temporal sclerosis (primarily affecting corticolimbic regions). The performance pattern of PLWH resembled that of the HD group more than the epilepsy group, reflecting a greater retrieval deficit than encoding deficit (Doyle et al. 2019); yet, the HIV group did not differ significantly from either the HD or epilepsy group. These results suggest that PLWH may have encoding and consolidation difficulties in addition to retrieval deficits.

Identifying patterns of neural correlates of the episodic memory deficit in PLWH and other people living with other diseases affecting frontostriatal systems could reveal relevant component cognitive processes underlying these deficits. Brain volume deficits associated with HIV infection and identified with imaging include frontal and limbic regions (Thompson et al. 2005; Pfefferbaum et al. 2018), which have been associated with different component processes of memory. Medial temporal lobe structures, in particular the hippocampus and surrounding structures, have historically been associated with episodic memory processes (Scoville and Milner 1957; Squire and Zola 1998), especially those of consolidation and retrieval, whereas extra-hippocampal structures including orbitofrontal and lateral frontal cortices, have been associated with encoding (Duarte et al. 2010; Ritchey et al. 2013; Wing et al. 2015). We previously identified a selective relation between episodic memory and orbitofrontal volume in individuals with alcohol use disorder that was salient in those comorbid for a drug diagnosis history (Fama et al. 2019). The relevance of the orbitofrontal region to encoding processes has been reported for over two decades in both non-human primate (Meunier et al. 1997) and human (Frey and Petrides 2000, 2002) functional imaging studies. Furthermore, rich connections exist between orbitofrontal and medial temporal regions (Frey and Petrides 2002), suggesting that these regions may be nodes of a neural system relevant to episodic memory function. Accordingly, in PD, both smaller limbic (medial temporal lobe) and frontostriatal (including superior frontal and pars orbitalis) volumes have been related to poorer recall (Filoteo et al. 2014).

The neural mechanism underlying the episodic memory deficit observed in PLWH remains unclear. It may be that the component processes of episodic memory in PLWH, especially with advancing age, may include those subserving both encoding and retrieval, and these deficits may not be simply secondary to executive dysfunction (Maki et al. 2009). Comparison of episodic memory processes in HIV infection compared with PD could distinguish component cognitive processes and neural correlates relevant to learning and retrieval of new information in conditions that affect frontostriatal systems. Episodic memory deficits are both common and relevant to everyday life including diminished activities of daily living and lower health-related quality of life in HIV (Osowiecki et al. 2000) and PD (Vasconcellos et al. 2019).

Herein, we tested the hypotheses that (1) both PLWH and people with PD would demonstrate episodic memory deficits compared with control participants, (2) executive function performance would not solely account for the episodic memory deficits observed in PLWH and individuals with PD, and (3) selective frontal volumes and limbic volumes would be associated with learning and retrieval (recall and recognition) in HIV and PD.

## Methods

### Participants

Participants included 42 HIV seropositive individuals (HIV: age 47–78 years; 25 men and 17 women), 41 individuals who met criteria for mild to moderate idiopathic Parkinson’s disease (PD: age 49–79 years; 26 men and 15 women), and 37 healthy control individuals (CTRL: age 45–77 years; 19 men and 18 women). Participants with HIV were recruited from community physicians and HIV treatment facilities. Participants with PD were recruited through regional PD-related events, the Michael J. Fox trial finder, and the Stanford University Neurology Clinic. CTRL participants were recruited through web-postings and flyers distributed throughout the local community. The Institutional Review Boards of Stanford University and SRI International approved all study procedures, and research was completed in accordance with the Declaration of Helsinki and its later amendments. All participants gave written informed consent at the beginning of the study and received a modest financial stipend for their time.

As we described previously (Müller-Oehring et al. 2021), all participants were assessed using the Structured Clinical Interview for DSM-IV (SCID-IV) (First et al. 1998). Each person with PD underwent a neurological examination by a board-certified neurologist. HIV serostatus was confirmed via blood testing. All participants with HIV were on a continuous regimen of ART for at least 2 months prior to testing. On average, ART adherence was 95% over the previous month prior to testing. Lifetime alcohol consumption (kg) was calculated for all participants using a semi-structured timeline follow-back interview (Skinner and Sheu 1982). Severity of depressive symptoms was assessed with the Beck Depression Inventory-II (Beck et al. 1996).

Participants were excluded if they had fewer than 8 years of education or a history of psychiatric (e.g., schizophrenia or bipolar disorder), neurological (other than PD), or medical (e.g., stroke, uncontrolled diabetes) condition potentially affecting the CNS other than HIV or had MRI contraindications. General current cognitive performance was assessed with the Dementia Rating Scale-2 (DRS-2) (Jurica et al. 2001) and a cutoff score for control participants was set at 136/144 (Springate et al. 2014).

Inclusion of participants with mild to moderate PD was determined by a neurologist and was based on a disease duration ≥ 2 years, Hoehn and Yahr stage < 4 off-dopaminergic medication (Hoehn and Yahr 1967; Hoehn and Yahr 1967), and improvement on medication assessed by the Movement Disorder Society–UPDRS part III scores (Goetz et al. 2008). Participants with PD were on dopaminergic medication during neuropsychological testing. No PD participant had neurosurgery as PD treatment.

Participants with HIV were more likely than those with PD or controls to have a history of past substance use. Past history of alcohol use disorder was documented in 13 HIV and 2 PD participants and were in full remission (range 3.6–46 years). Participants who reported drug use were in full remission for at least 3½ years for all substances, except cannabis (*n* = 4 HIV) and nicotine (*n* = 15 HIV, *n* = 2 CTRL).

Groups differed on age, education, and SES, with the PD group being older than the HIV and CTRL groups and the HIV group having fewer years of education and lower SES than the PD or CTRL groups (Table 1). All participants with HIV or PD scored above a 124 on the DRS (cutoff score for dementia screen), with the exception of 1 PLWH who scored 114/144. The HIV and PD groups differed from one another on time since disease diagnosis, with a number of HIV participants having been diagnosed decades ago. Participants with HIV were on a stable antiretroviral treatment (ART) regimen and had undetectable viral loads, average CD4^+^ T-cell counts of 747.4 cells/mm^3^ (median = 788 cells/mm^3^, range = 221–1576 cells/mm^3^); 27 HIV participants had been diagnosed with AIDS (i.e., having had an AIDS-defining event and/or a CD4^+^ T-cell count less than 200 cells/mm^3^ at any time since HIV infection).

**Table 1:** Demographic and disease-related variables: mean, standard deviation, and range

	Sex (F/M)	Age	Education	DRS	SES	Disease duration	CD4	Nadir CD4	LEDD (mg)	UPDRS-III
HIV (*n* = 42)	17/25	59.95	14.19	136.69	37.67	24.66	747.4	181.9		4.1
		(6.98)	(2.23)	(5.89)	(14.57)	(7.90)	296.4	156.3	na	4.3
		47–78	10–19	114–144	11–69	6–40	221–1576	0–600		0–19
PD (*n* = 41)	15/26	66.05	16.71	138.48	20.37	4.71			596.49	15.8
		(7.52)	(2.04)	(3.62)	(8.21)	(3.00)	na	na	(294.58)	7.7
		49–79	12–21	129–144	11–40	1–14			100–1440	5–36
CTRL (*n* = 37)	18/19	61.38	16.43	140.84	22.68					1.9
		(8.77)	(2.47)	(1.95)	(10.49)	na	na	na	na	2.7
		45–77	12–21	137–144	11–47					0–11
ANOVA/*t* test		*F*(2, 117) = 6.96, *p* = 0.0014	*F*(2, 117) = 15.56, *p* < 0.0001	*F*(2, 116) = 9.49, *p* = 0.0002	*F*(2,117) = 27.71, *p* < 0.0001	*t*(81) = 15.13, *p* < 0.0001				*F*(2, 113) = 75.14, *p* < 0.0001
Post-hoc *t* tests		PD > HIV, CTRL	HIV < PD, CTRL	HIV, PD < CTRL	HIV > PD, CTRL	HIV > PD				PD > HIV, CTRL

For Sex Likelihood Ratio Chi Square = 1.199, *p* = 0.550, ns; HIV group- *n* = 42 except CD4 *n* = 37, Nadir CD4 *n* = 36, UPDRS-III *n* = 40; PD group *n* = 41 except DRS *n* = 40, LEDD *n* = 40, UPDRS-III *n* = 40

Participant groups differed in their racial makeup. In the control group, 1 participant out of the group of 37 participants identified as Hispanic, 1 participant identified as Native American, 4 identified as Asian, 5 identified as Black, 1 identified as Pacific Islander, and 25 participants identified as white. In the HIV group, 3 participants out of the group of 42 participants identified as Hispanic, 1 participant identified as Native American, 17 identified as Black, 1 identified as Pacific Islander, and 26 participants identified as white. In the PD group, 3 participants out of the group of 41 participants identified as Hispanic, 1 identified as Asian, and 25 participants identified as white. Thus, the HIV group had a higher proportion of participants who identified as Black compared with the PD or control groups; the PD group had a higher proportion of participants who identified as white than either the HIV or control groups; and the control group had a higher proportion of participants who identified as Asian than the HIV or PD groups.

### Episodic verbal learning and memory measure

Episodic memory was assessed with the California Verbal Learning Test-II (CVLT-II) (Delis et al. 2000). The CVLT-II comprises 5 learning trials, each trial consisting of 16 words with 4 words each derived from 4 different categories (List A), followed by a single learning trial (List B) of a different list of 16 words. There is a short-delay free recall and category cued recall of List A immediately after List B and then a long-delay free recall and category cued recall of List A after a 30-min delay from the end of the 5 learning trials. Scores for number of words learned over 5 trials, free recall of List B, and number of words recalled on short- and long-delay free and cued conditions were dependent variables. In addition, retention scores, based on Trial 5 performance, were derived for free recall and cued recall performance. Specifically, Trial 5 score (number of correct words recalled on the last learning trial) was subtracted from free and cued recall score at short- and long-delay trials. This calculated difference score was then age- and education-corrected based on the CTRL group and presented as Z-scores.

### Executive function composite score

An age- and educated-corrected composite Z-score was calculated based on the average score of the Stroop Color-Word naming trial (Golden and Freshwater 2002) and Digit Span backwards from the Wechsler Memory Scale—Revised (WMS-R) (Wechsler 1987), as previously reported (Muller-Oehring et al. 2021). The Stroop Color-Word naming trial assesses inhibitory control, and Digit Span backwards assesses working memory and sequencing. These executive function domain scores were used to examine the potential influence of executive function performance on CVLT component memory scores.

### MRI acquisition and analysis

The methods have been described previously (Fama et al. 2019; Fama et al. 2021) and are summarized here. MRI data were acquired on 3 Tesla GE whole body MR systems (General Electric Healthcare, Waukesha, WI) using an 8-channel phased-array head coil. T1-weighted Inversion-Recovery Prepared SPGR images (TR = 6.55/5.92 ms, TE = 1.56/1.93 ms, TI = 300/300 ms, matrix = 256 × 256, thick = 1.25 mm, skip = 0 mm, 124 slices) based on an axial structural sequence was used for volumetric analysis. Drift was corrected by adjusting scanner calibration parameters when necessary to maintain spatial stability within manufacturer guidelines, and routine phantom data were used to evaluate spatial fidelity.

Preprocessing of the T1-weighted MRI data involved noise removal (Coupe et al. 2008), correcting field inhomogeneity via N4ITK (Tustison et al. 2011), and segmenting the brain mask by majority voting (Rohlfing et al. 2004). The voting was performed with respect to the maps generated by separately applying FSL BET (Smith 2002), AFNI 3dSkullStrip (Cox 1996), FreeSurfer mri_gcut (Sadananthan et al. 2010), and the Robust Brain Extraction (ROBEX) method (Iglesias et al. 2011) to the bias and non-bias corrected T1-weighted MRIs.

**Fig. 1:**
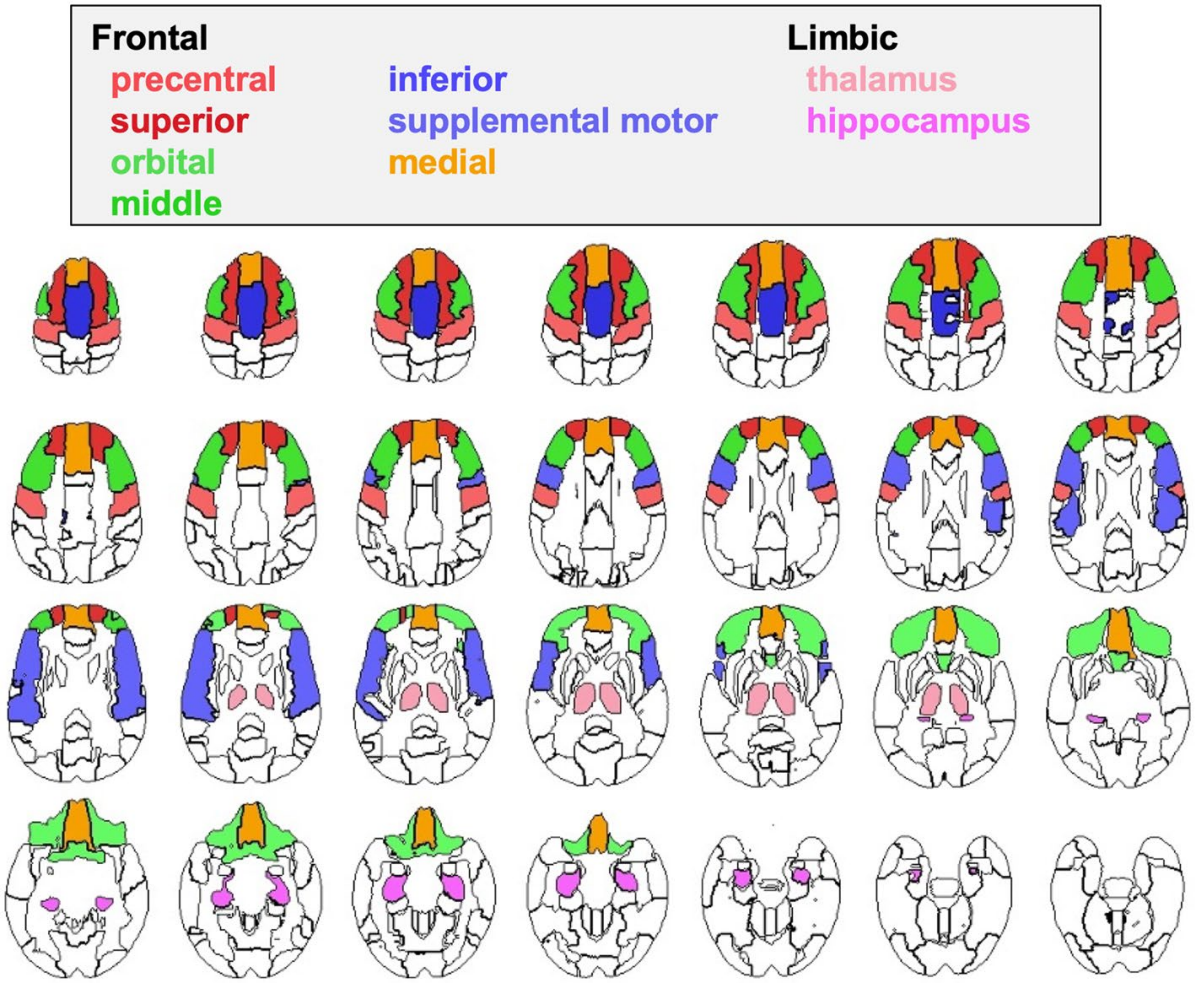
Color-coded atlas identifying the regional volumes of the frontal cortices, hippocampus, and thalamus

Brain tissue segmentation (gray matter, white matter, and cerebrospinal fluid) of the skull-stripped T1-weighted MRI was generated via Atropos (Avants et al. 2011). The label map was further parcellated into the regions defined by the SRI24 atlas (Rohlfing et al. 2010) by non-rigidly registering the atlas to the MRI via ANTS (Avants et al. 2008). Frontal gray matter was parcellated into seven regions of interest: precentral, superior, orbital, middle, inferior, supplemental motor, and medial (Fig. 1). Based on our hypotheses we also measured the hippocampus and thalamus. Brain volumes were age- and head-sized corrected based on 238 control participants (19–86 years). Automatic labeling was always visually inspected for accuracy by a trained research scientist.

### Statistical analysis

All verbal learning and memory scores were age- and education-corrected based on the CTRL group using regression analyses, such that the mean score and standard deviation of the CTRL group was equal to 0 ± 1. Z-scores allowed for direct comparison across individual test scores within and between groups. Age- and education-corrected Z-scores were also computed for the executive function tests, and an average of these scores comprised the Executive Function Domain score (Müller-Oehring et al. 2021). Not all test scores and brain volumes were normally distributed (assessed with Shapiro–Wilk test) or demonstrated similar variance between groups (assessed with Levene test); thus nonparametric statistics are reported. Z-scores for short- and long-delay trials were averaged such that there were four delayed memory scores: (1) free recall, (2) cued recall, (3) retention—free recall, and (4) retention—cued recall. Spearman’s rho were calculated to examine the relations between verbal learning and memory scores and brain volumes. A false discovery rate correction (Benjamini and Hochberg 1995) was employed for brain–behavior correlational analyses based on 6 comparisons (learning and memory scores) for the a priori hypotheses (critical alpha level = 0.017; based on one-tailed tests). Cohen’s *d*, a measure of effect size, was calculated for group differences and Cohen’s *q* was calculated to test the effect size (strength of the relations) between Spearman’s rho for the HIV and PD groups. Planned secondary analyses tested for differences in brain–behavior relations in individuals with HIV with and without a history of a drug diagnosis and those with and without a history of an alcohol use diagnosis.

## Results

### Group differences on episodic memory scores (CVLT)

Means, standard deviations, and nonparametric statistics (Kruskal–Wallis and Mann–Whitney tests) are presented in Table 2 and Supplemental Table 1 for all learning, recall, and recognition scores. Raw scores (not adjusted for age or education) for individual CVLT variables are presented in Table 3.

**Table 2:** CVLT-II age- and education-corrected Z-scores (mean, sd)

	HIV (H)	PD	CTRL (C)	Kruskal–Wallis test	Cohen’s d	Post-hoc comparisons	Cohen’s d
Total trials 1–5	− 0.746 (1.46)	– 0.577 (1.37)	− 0.005 (1.01)	*H*(2) = 7.58, *p *= 0.023*	0.448	H<C: Z = 2.63, *p *= 0.009*	0.619
						PD<C: Z = 2.08, *p *= 0.037*	0.485
Free recall	– 0.857 (1.40)	– 0.664 (1.22)	− 0.003 (.98)	*H*(2) = 9.61, *p *= 0.008*	0.527	H<C: Z = 2.86, *p *= 0.004*	0.681
						PD<C: Z = 2.43, *p *= 0.015*	0.573
Cued recall	– 0.967 (1.45)	– 0.852 (1.35)	− 0.004 (.98)	*H*(2) = 11.40, *p *= 0.003*	0.591	H<C: Z = 3.01, *p *= 0.002*	0.720
						PD<C: Z = 2.75, *p *=0.006*	0.656
Recognition	– 0.328 (1.54)	– 0.304 (0.99)	− 0.005 (1.02)	*H*(2) = 2.45, *p *= 0.293	0.125	ns	
Retention—free recall	– 0.439 (1.30)	– 0.122 (1.19)	0.002 (0.93)	*H*(2) = 2.87, *p *= 0.239	0.173	ns	
Retention—cued recall	– 0.416 (1.22)	– 0.206 (1.41)	0.002 (0.95)	*H*(2) = 2.64, *p *= 0.268	0.148	ns	

*Denotes significant group difference

**Table 3:** CVLT raw scores (mean, sd)

	HIV	PD	CTRL
Trial 1 (max = 16)	5.14 (2.41)	5.41 (1.90)	6.22 (2.06)
Trial 2 (max = 16)	7.88 (2.89)	8.83 (2.77)	9.22 (2.24)
Trial 3 (max = 16)	9.29 (2.83)	10.24 (2.92)	11.32 (2.26)
Trial 4 (max = 16)	10.02 (3.14)	10.68 (2.79)	12.22 (2.31)
Trial 5 (max = 16)	10.79 (2.83)	11.32 (2.69)	12.97 (2.43)
Total Trials 1–5 (max = 80)	43.12 (12.53)	46.49 (11.61)	51.95 (9.13)
List B (max = 16)	4.48 (2.22)	4.66 (1.93)	5.46 (1.95)
Short-delay Free Recall (max = 16)	8.98 (3.60)	9.85 (3.52)	11.84 (3.24)
Short-delay Cued Recall (max = 16)	10.07 (3.13)	11.15 (2.97)	12.86 (2.43)
Long-delay Free Recall (max = 16)	9.07 (3.81)	10.44 (3.43)	12.43 (2.71)
Long-delay Cued Recall (max = 16)	10.26 (3.28)	10.80 (3.27)	13.11 (2.38)
Recognition (max = 16)	14.55 (1.74)	14.85 (1.13)	15.24 (1.23)

**Fig. 2:**
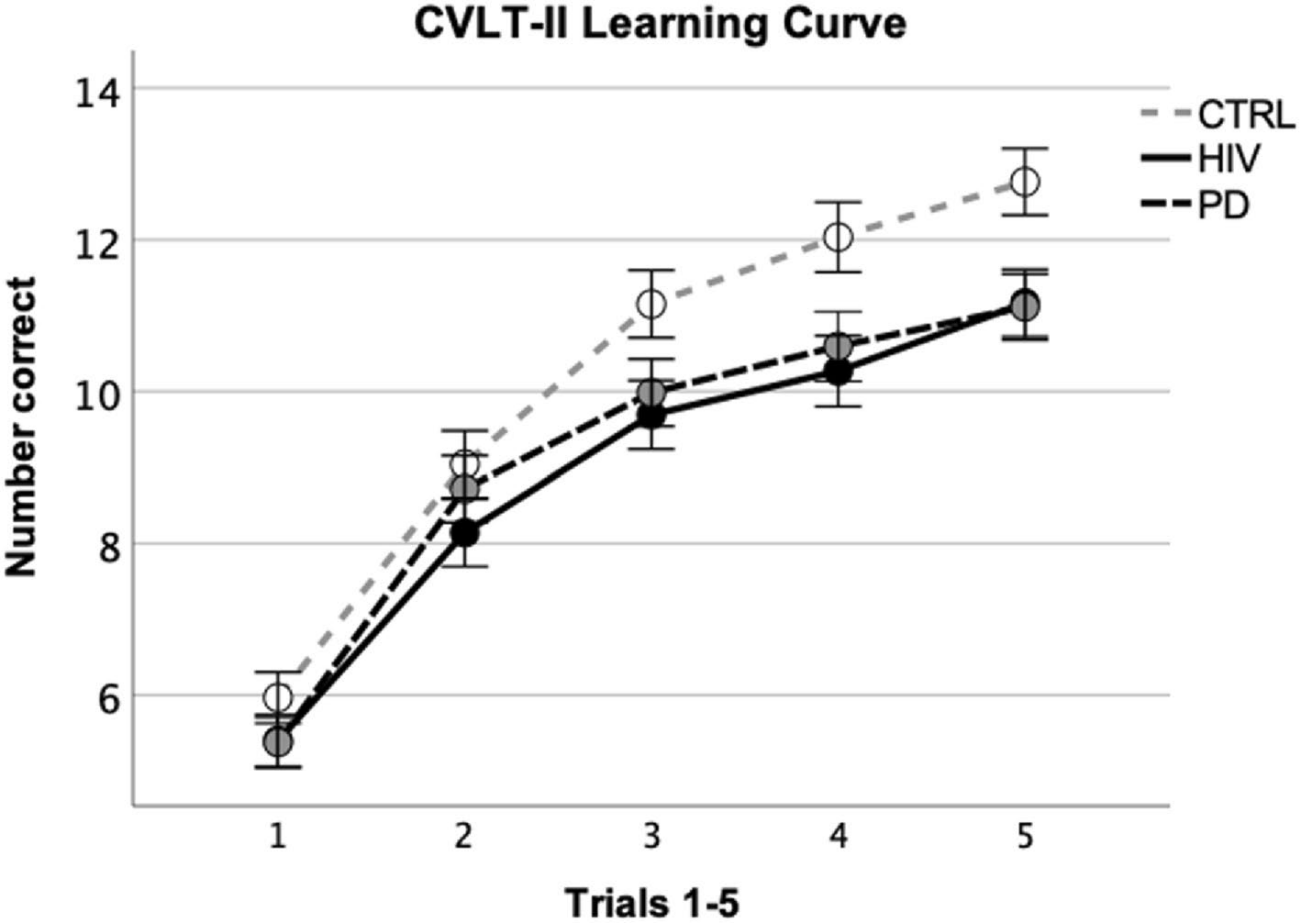
Graph depicting Z-scores over learning trials 1–5 in the HIV, PD, and CTRL groups

Kruskal–Wallis analyses indicated group differences for number of words learned on trial 4 [*H*(2) = 6.94, *p* = 0.031 Cohen’s d = 0.420] and trial 5 [*H*(2) = 8.65, *p* = 0.013, Cohen’s d = 0.491] (Fig. 2). Groups also differed on total number of words recalled over the 5 learning trials (Trials 1–5) [*H*(2) = 7.58, *p* = 0.023] and number of words recalled on free recall [*H*(2) = 9.61, *p* = 0.008] and cued recall trials [*H*(2) = 11.40, *p* = 0.003] (Table 2). Groups did not differ on recognition score. Post-hoc analyses indicated that the HIV and PD groups scored lower than the CTRL group on total words recalled over learning trials (Trials 1–5), free recall, and cued recall. No group differences were observed on delayed free and cued recall when scores were adjusted for the number of words recalled on the last learning trial (retention—free recall, retention—cued recall).

Within-group comparisons indicated that all 3 groups recalled more words on cued recall than free recall: CTRL *t*(36) = 5.18, *p* < 0.0001, HIV *t*(41) = 6.14, *p* < 0.0001, PD *t*(40) = 4.76, *p* < 0.0001.

### Group differences in brain volumes

For thalamic volume [*H*(2) = 9.18, *p* = 0.010, Cohen’s d = 0.511), the HIV group had significantly smaller volume than the PD group (*Z* = 2.98, *p* = 0.003, Cohen’s d = 0.693) (Table 4). Groups did not differ on hippocampal volume. Although groups showed a modest difference on total frontal volume [*H*(2) = 4.93, *p* = 0.085, Cohen’s d = 0.321), no group difference was observed for any of the regional frontal volumes.

**Table 4:** Age- and head-size corrected regional brain volumes (mean, sd)

Volumes (cc)	HIV (*n* = 42)	PD (*n* = 41)	CTRL (*n* = 37)	Kruskal–Wallis test	Cohen’s d
Frontal					
Total	131.4 (9.7)	135.0 (7.9)	136.1 (5.8)	*H*(2) = 4.93, *p* =0 .085	0.321
Precentral	15.2 (2.1)	15.8 (2.0)	16.0 (1.8)	*H*(2) = 2.20, *p* = 0.332	0.084
Superior	17.7 (1.7)	17.7 (1.3)	18.2 (1.8)	*H*(2) = 2.96, *p* = 0.227	0.182
Orbital	23.8 (1.9)	24.0 (2.0)	24.3 (1.7)	*H*(2) = 1.52, *p* = 0.467	0.128
Middle	22.1 (2.9)	23.4 (2.5)	23.3 (2.5)	*H*(2) = 3.75, *p* = 0.153	0.247
Inferior	21.6 (1.9)	22.2 (2.1)	22.3 (1.4)	*H*(2) = 2.77, *p* = 0.250	0.163
Supplemental motor	10.1 (1.6)	9.9 (1.3)	10.2 (1.2)	*H*(2) = 0.97, *p* = 0.615	0.188
Medial	21.3 (2.2)	21.7 (1.8)	21.9 (1.5)	*H*(2) = 1.41, *p* = . 0.495	0.143
Hippocampus	8.1 (0.7)	8.2 (0.7)	8.1 (0.6)	*H*(2) = 0.13, *p* = 0.950	0.255
Thalamus	10.7 (1.3)	11.4 (0.7)	11.0 (0.8)	*H*(2) = 9.18, *p* = 0.010 HIV < PD: Z = 2.98, *p* = 0.003	0.511* 0.693*

*Denotes significant group difference

### Brain–behavior correlations

**Fig. 3:**
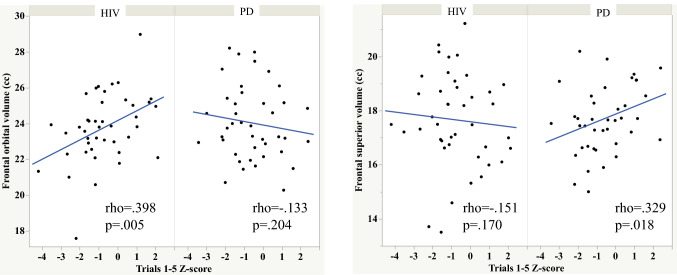
Scatterplots depicting the double dissociation observed between frontal volumes and number of words recalled over learning trials 1–5: frontal orbital volume was related to learning over trials in the HIV group, whereas frontal superior volume was related to learning over trials in the PD group

An adjusted significance value of *p* ≤ 0.017 based on a false discovery rate correction for 6 comparisons (one-tailed) was employed to the Spearman’s rho analyses. In the HIV group, total number of words recalled over the five learning trials (Trials 1–5), number of words recalled on free recall, and number of words recalled on cued recall were correlated with frontal orbital volumes (Table 5). By contrast, total number of words recalled over the five learning trials (Trials 1–5) correlated with frontal superior volume in the PD group (*p* value = 0.0177) (Fig. 3).

**Table 5:** Spearman’s rho correlations between CVLT scores and brain volumes

	Trials 1–5	Free recall	Cued recall	Recognition	Retention—free recall	Retention—cued recall
HIV Group						
Frontal						
Precentral	− 0.108	− 0.116	− 0.135	0.082	− 0.079	− 0.140
Superior	− 0.151	− 0.130	− 0.106	− 0.107	− 0.148	− 0.180
Orbital	0.398*	0.423*	0.429*	0.180	0.168	0.123
Middle	− 0.058	− 0.094	− 0.042	0.023	− 0.101	− 0.052
Inferior	0.040	0.089	0.040	− 0.007	0.107	0.030
Supplemental motor	0.131	0.046	0.059	0.165	− 0.076	− 0.090
Medial	0.064	0.030	0.032	− 0.005	0.018	− 0.003
Hippocampus	− 0.020	0.017	− 0.031	− 0.099	0.131	0.142
Thalamus	− 0.112	− 0.124	− 0.206	− 0.226	− 0.019	− 0.088
PD Group						
Frontal volumes						
Precentral	0.308	0.247	0.168	− 0.019	0.004	− 0.141
Superior	*0.329*^#^	0.201	0.177	0.025	− 0.108	− 0.159
Orbital	− 0.133	− 0.093	− 0.050	− 0.077	0.061	0.137
Middle	0.088	− 0.008	0.012	− 0.003	− 0.194	− 0.190
Inferior	0.271	0.197	0.208	0.125	0.047	0.118
Supplemental motor	0.059	0.136	0.128	− 0.132	0.137	0.074
Medial	0.053	0.008	− 0.036	− 0.252	− 0.200	− 0.169
Hippocampus	− 0.023	0.076	0.032	0.120	0.338*	0.281
Thalamus	0.282	0.189	0.204	0.355*	0.043	0.037

*Denotes significant correlation at *p* < 0.017 FDR adjusted for multiple comparisons

^#^Spearman’s rho = 0.329, *p* = 0.0177

**Fig. 4:**
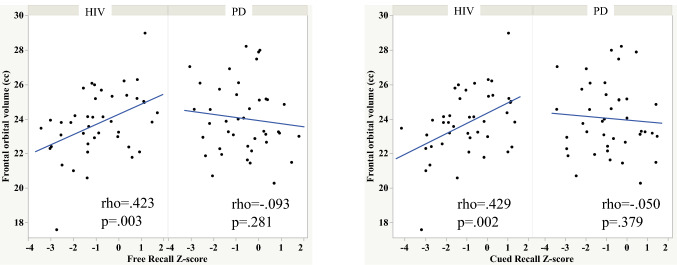
Scatterplots depicting the relationship between orbitofrontal volume and free and cued recall in the HIV and PD groups

**Fig. 5:**
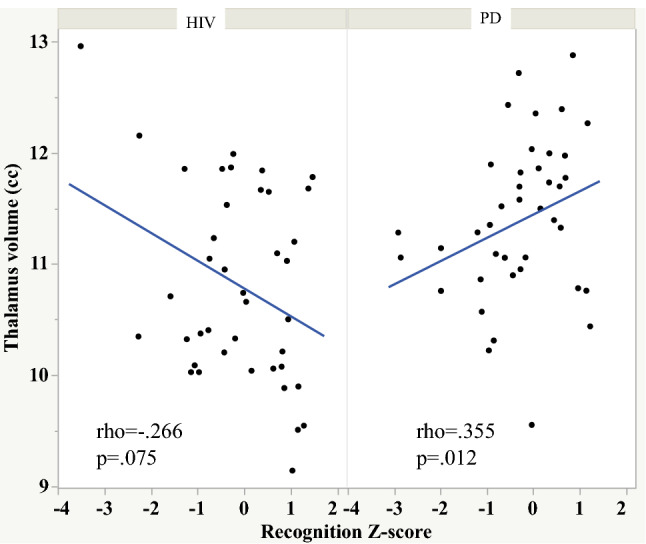
Scatterplots depicting the relationship between thalamic volume and recognition memory score in the HIV and PD groups. Note: there were 2 HIV participants who had outlying scores and were excluded from the scatterplot. Analyses with and without these HIV individuals resulted in similar Spearman’s rho and *p* values: HIV (*n* = 42) rho = − 0.226, *p* = 0.150; HIV (*n* = 40) rho = − 0.240, *p* = 0.135

**Fig. 6:**
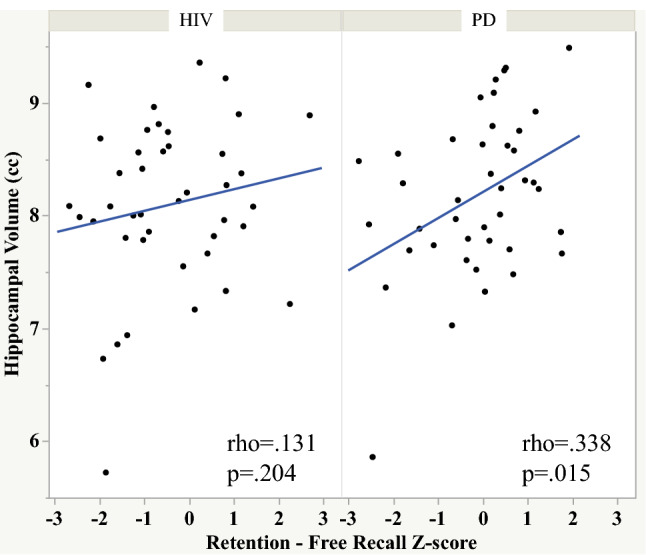
Scatterplots depicting the relationship between hippocampal volume and retention—free recall score in the HIV and PD groups

Free and cued recall also correlated with frontal orbital volume in the HIV group (Fig. 4). By contrast, recognition score correlated with thalamic volume, and the retention—free recall score correlated with hippocampal volume in the PD group (Figs. 5 and 6).

To test whether the HIV and PD groups differed in strength of relationships between select memory scores and brain volumes we calculated Cohen’s q. A large effect was noted between the Spearman’s rho for the HIV and PD groups depicting the relations between orbitofrontal volume and total number of words recalled over the learning trials (Cohen’s q = 0.555), free recall (Cohen’s q = 0.545), and cued recall (Cohen’s q = 0.509) scores. A large effect was also noted between the PD and HIV groups for the relation between thalamic volume and recognition score (Cohen’s q = 0.601). A small effect was noted between the PD and HIV groups for the relation between hippocampal volume and retention—free recall score (Cohen’s q = 0.220).

### Correlations between HIV- and PD-related disease parameters and episodic memory scores

Disease duration was not correlated with any of the CVLT scores in the HIV or PD groups (Supplemental Table 3). CD4^+^ cell count and CD4^+^ nadir count were not significantly correlated with any of the CVLT scores in the HIV group. UPDRS-III score was not correlated with any of the CVLT scores in the HIV or PD group.

### Correlations between memory scores and frontal orbital volume in HIV with and without a history of a drug or alcohol use diagnosis

To test whether the relation between CVLT scores and frontal orbital volumes was not due to a history of a substance use diagnosis, Spearman rhos were calculated for the HIV participants who did not have a history of a drug diagnosis (*n* = 20), excluding those HIV participants who had a history of a drug diagnosis (*n* = 22) (Supplemental Table 2). Relations based on frontal orbital volumes remained significant in the subgroup of HIV participants without a history of a drug diagnosis for words learned over Trials 1–5, free recall, and cued recall scores.

Next, Spearman rhos were calculated separately for the HIV subgroup who did not have a history of an alcohol use diagnosis (*n* = 32), excluding those HIV participants who did have a history of an alcohol use diagnosis (*n* = 10) (Supplemental Table 2). Again, significant relationships were observed between frontal orbital volume and words learned over Trials 1–5, free recall, and cued recall scores in the subgroup of HIV participants without a history of an alcohol use diagnosis.

### Correlations between executive function domain score and episodic memory scores

The executive function domain score was significantly correlated with total words recalled over Trials 1–5 (rho = 0.40, *p* = 0.004) in the HIV group. Executive function domain score was not significantly correlated with any of the free recall, cued recall, or recognition scores in the HIV group.

A somewhat different pattern of relation was observed in the PD group, with executive function domain score significantly correlated with number of words recalled on free recall (rho = 0.28, *p* = 0.019), cued recall (rho = 0.42, *p* = 0.0032)], and retention-cued recall (rho = 0.35, *p* = 0.012).

Stepwise multiple regression analyses tested scores that were related to both executive function domain score and regional brain volumes to examine whether either, both, or neither were unique predictive factors, and interaction terms were tested if they were different predictors for the clinical groups (Table 6). Both frontal orbital volume and executive function score were correlated with Trials 1–5 and cued recall scores in the HIV group. Multiple regression analysis models indicated that the executive function score and the orbitofrontal volume-by-group interaction were independent predictors of these CVLT scores, with executive function accounting for 17.2% and the orbital frontal volume-by-group interaction adding 7.3%, all variables together explaining 25.8% of the variance in the Trials 1–5 score; and executive function accounted for 12.4% and the orbital frontal volume-by-group interaction added 7.6%, with all variables together explaining 25.4% of the variance in the cued recall score.

**Table 6:** Hierarchical regression analyses for CVLT Z-scores for trials 1–5, cued recall, and retention—cued recall

CVLT measures	Predictor	*t*	*p*	Step	*R*^2^ change	*F* change	*p* change	*R*^2^	*F*	*p*
Trials 1–5	EF	− 2.400	**0.019**	1	**0.172**	16.612	** < .001**	0.172	16.612	< .001
	IA EF-by-group	0.441	0.661	2	0.001	0.073	0.787	0.173	8.247	< .001
	OFC	1.027	0.307	3	0.012	1.177	0.281	0.185	5.902	0.001
	IA OFC-by-group	− 2.746	**0.007**	**4**	**0.073**	7.543	**0.007**	**0.258**	**6.684**	** < .001**
Cued Recall	EF	− 3.243	**0.002**	1	**0.124**	11.308	**0.001**	0.124	11.308	0.001
	IA EF-by-group	1.816	0.073	2	0.027	2.477	0.12	0.15	6.997	0.002
	OFC	0.645	0.521	3	0.028	2.643	0.108	0.178	5.642	0.001
	IA OFC-by-group	− 2.797	**0.007**	**4**	**0.076**	7.825	**0.007**	**0.254**	**6.558**	** < .001**
Retention – Cued Recall	EF	0.612	0.542	1	0.006	0.445	0.507	0.006	0.445	0.507
	IA EF-by-group	− 2.305	**0.024**	2	**0.057**	4.835	**0.031**	0.063	2.65	0.077
	hippocampus	− 0.824	0.413	3	**0.057**	5.017	**0.028**	0.12	3.529	0.019
	IA hippo-by-group	− 1.085	0.281	**4**	0.013	1.177	0.281	**0.133**	**2.947**	**0.025**

*t* and *p* values represent the full multivariate regression model at step 4. Bolded values denote significance at *p* < 0.05 of a variable and interaction (IA) term in the full regression model

In the PD group, both hippocampal volume and executive function score were correlated with cued retention. Multiple regression indicated that the executive function-by-group interaction and hippocampal volume were independent predictors of cued retention, with the executive function-by-group interaction accounting for 6.3% and hippocampal volume adding 8.8%, all variables together explaining 13.3% of the variance.

## Discussion

These findings are consistent with previous studies reporting episodic verbal memory deficits in HIV infection (Fama et al. 2009, 2016; Tan et al. 2013; Woods et al. 2009; Peavy et al. 1994) and PD (Pirogovsky-Turk et al. 2015; Das et al. 2019) and introduce brain substrates associated with component processes of episodic memory in HIV and PD, which affect fronto-subcortical regions. This study also extends previous reports by demonstrating that although the severity of episodic memory deficits were similar in the HIV and PD groups, dissociable neural correlates differentiated the two diseases. Specifically, a double dissociation indicated that diminished learning over trials was related to smaller orbitofrontal volume in the HIV but not the PD group, whereas diminished learning over trials was related to smaller superior frontal volume in the PD but not the HIV group. Other dissociations were also observed, including (1) poorer free and cued recall related to smaller orbitofrontal volume in HIV but not PD, (2) poorer recognition related to smaller thalamic volume in PD but not HIV, and (3) poorer retention on free recall related to smaller hippocampal volume in PD but not HIV. Such brain–behavior dissociations have implications in the conceptualization of the mechanisms underlying episodic memory deficits in these diseases.

In addition to showing relations between episodic memory and executive function performance in the HIV and PD groups, we demonstrated that standard tests of executive function were independent predictors of episodic memory performance in both groups. Notwithstanding, there was also evidence of extra-frontostriatal substrates involvement in episodic memory performance. Specifically, the relations based on orbitofrontal volume in HIV and superior frontal volume in PD emphasize neural regions believed to be associated with learning and encoding. Similarly, the relation with thalamic volume highlights an area associated with recollection and familiarity, acting as a hub between medial prefrontal and hippocampal regions (Thielen et al. 2015), and hippocampal volume, an area associated with consolidation of new information (Scoville and Milner 1957; Squire and Zola 1998), with retention of information in the PD group. Thus, within the mnemonic network for episodic memory processes, despite a similar deficit phenotype, the HIV and PD groups exhibited different pathogenic patterns—with PD-associated vulnerability of the hippocampus for retention of learned information (see, e.g., Tambini and Davachi 2013) and neural substrates beyond the role of the hippocampus including HIV-associated vulnerability in the orbitofrontal cortex, an area with bi-directional connections to medial temporal regions, relevant to learning and encoding (Frey and Petrides 2002; Li 2022) and PD-associated vulnerability in the superior frontal cortex, a region associated with top-down control for learning and encoding of complex material (Ritchey et al. 2013; Wing et al. 2015).

There are reports that episodic memory deficits in early PD may be prodromal for further cognitive decline (Broeders et al. 2013; Hoogland et al. 2017; Tsiouris et al. 2020). Support for this speculation derives from involvement of posterior cortices including temporal and parietal regions, in addition to the frontostriatal neuropathology primarily responsible for the motor-related and slowed information processing speed characteristic of PD (Montaser-Kouhsari et al. 2022). Based on these brain–behavior relations, the episodic memory deficit associated with orbitofrontal volume in HIV may reflect extra-frontostriatal, namely, fronto-limbic dysfunction, which may also be prognostic of further cognitive decline with disease progression (Brown et al. 2022). Different nodes within the fronto-limbic mnemonic circuitry may underlie the mutual verbal episodic memory deficits observed in individuals with HIV and PD.

There are study limitations that need to be noted. The sample size of our groups was modest, and the HIV group was relatively younger and had fewer years of education than the PD group and controls. Racial makeup of our clinical groups differed, with a higher percentage of white participants in the PD group and a higher percentage of Black participants in the HIV group compared with each other and the control group. Despite these demographic differences, the HIV and PD groups did not differ in performance on the memory tests. Furthermore, although there were a number of different episodic memory scores examined, they all derived from a single list learning measure, and although we examined the relevance of executive function to memory processes, we did not examine other possibly relevant component cognitive processes, such as sustained attention. Finally, the hippocampus and thalamus volumes were based on the entire structure and not subregional nuclei, which may have afforded a more focused substrate of selective memory processes.

In conclusion, this study identified selective and dissociable neural correlates of episodic memory processes in HIV infection and PD. Taken together, the brain–behavior relations revealed in HIV and PD contribute to the identification of mechanistic factors underlying deficits in different component processes of episodic memory. Thus, despite the common overarching deficit in episodic memory of these two diseases affecting striatal systems, examination of extra-striatal correlates for brain substrates of observed deficits revealed dissociable mechanisms underlying deficit performance.

## Supplementary Information

Below is the link to the electronic supplementary material.

Supplementary file1 (DOCX 29 KB)

## Data Availability

The data sets generated during and/or analyzed during the current study are available from the corresponding author upon reasonable request.
