# Supplementary material for: Episodic memory deficit in HIV infection: common phenotype with Parkinson’s disease, different neural substrates
Source: Brain Struct Funct. 2023 Apr 18;228(3-4):845–58. doi: 10.1007/s00429-023-02626-x (PMC10147801; doi:10.1007/s00429-023-02626-x)
Supplement: Supplementary file 1 — Supplementary file1 (DOCX 29 KB) [file 429_2023_2626_MOESM1_ESM.docx]

Episodic memory deficit in HIV infection: Common phenotype with Parkinson’s disease, different neuromechanism

Authors: Rosemary Fama, Eva M. Müller-Oehring, Taylor F. Levine, Edith V. Sullivan, Stephanie A. Sassoon, Priya Asok, Helen M. Brontë-Stewart, Kathleen L. Poston, Kilian M. Pohl, Adolf Pfefferbaum, and Tilman Schulte

SUPPLEMENTAL MATERIAL

Supplemental Table 1: CVLT-II age- and education-corrected Z Scores for individual scores: mean (sd)

Supplemental Table 2: Spearman Rho Correlations between orbitofrontal volume and CVLT-II Z-scores in the HIV subgroups with and without a history of a drug or AUD diagnosis

Supplemental Table 3: Spearman Rho Correlations between CVLT Z-scores and Disease-Related Variables in HIV and PD

| Supplementary Table 1:  CVLT-II age- and education-corrected Z Scores for individual scores: mean (sd) | | | | | |
| --- | --- | --- | --- | --- | --- |
|  | HIV | PD | CTRL | Kruskal-Wallis Test | Cohen's d |
| Trial 1 | -.370 | -.381 | -.004 | H(2)=4.692, p=.096 | .307 |
|  | (1.12) | (.93) | (1.02) |  |  |
| Trial 2 | -.451 | -.118 | -.006 | H(2)=4.992, p=.082 | .324 |
|  | (1.33) | (1.27) | (1.01) |  |  |
| Trial 3 | *-.655* | *-.477* | *.001* | H(2)=5.309, p=.07 | .341 |
|  | (1.35) | (1.40) | (1.01) |  |  |
| Trial 4 | -.786 | -.595 | -.003 | H(2)=6.941, p=.031 | .420 |
|  | (1.52) | (1.31) | (1.01) |  |  |
| Trial 5 | -.668 | -.692 | -.005 | H(2)=8.65, p=.013 | .491 |
|  | (1.23) | (1.89) | (1.02) |  |  |
| List B | -.325 | -.292 | -.005 | H(2)=2.177, p=.337 | .078 |
|  | (1.19) | (1.11) | (1.01) |  |  |
| Short-delay Free Recall | -.673 | -.614 | -.002 | H(2)=7.892, p=.019 | .461 |
|  | (1.22) | (1.12) | (1.01) |  |  |
| Short-delay Cued Recall | -.984 | -.677 | -.004 | H(2)=10.783, p=.005 | .570 |
|  | (1.42) | (1.29) | (1.01) |  |  |
| Long-delay Free Recall | -1.042 | -.714 | -.004 | H(2)=10.052, p=.007 | .544 |
|  | (1.66) | (1.39) | (1.01) |  |  |
| Long-delay Cued Recall | -.950 | -1.027 | -.003 | H(2)=11.283, p=.004 | .587 |
|  | (1.57) | (1.45) | (1.01) |  |  |
| Retention: Short-delay Free Recall | -.277 | -.159 | .003 | H(2)=1.447, p=.485 | .138 |
|  | (1.35) | (1.16) | (1.01) |  |  |
| Retention: Short-delay Cued Recall | -.478 | .018 | .000 | H(2)=5.05, p=.08 | .327 |
|  | (1.37) | (1.36) | (1.01) |  |  |
| Retention: Long-delay Free Recall | -.600 | -.085 | .000 | H(2)=4.098, p=.129 | .270 |
|  | (1.43) | (1.39) | (1.02) |  |  |
| Retention: Long-delay Cued Recall | -.354 | -.430 | .003 | H(2)=1.693, p=.429 | .103 |
|  | (1.27) | (1.55) | (1.02) |  |  |

| Supplementary Table 2:  Correlations between orbitofrontal volume and CVLT-II scores in the HIV subgroups with and without a history of a drug diagnosis or an AUD diagnosis | | | | | | | |
| --- | --- | --- | --- | --- | --- | --- | --- |
| HIV with no drug dx history (n=20) | Orbitofrontal Volume | | | HIV with no AUD history (n=32) | Orbitofrontal Volume | | |
|  | rho | p |  |  | rho | p |  |
| Trials 1-5 | *.496* | *.026* | ***** | Trials 1-5 | *.441* | *.012* | ***** |
| Free Recall | *.456* | *.044* | ***** | Free Recall | *.499* | *.004* | ***** |
| Cued Recall | *.543* | *.013* | ***** | Cued Recall | *.488* | *.005* | ***** |
| Recognition | .277 | .238 |  | Recognition | .161 | .380 |  |
| Retention: Free Recall | .326 | .160 |  | Retention: Free Recall | .213 | .243 |  |
| Retention: Cued Recall | .248 | .292 |  | Retention: Cued Recall | .213 | .243 |  |
| HIV with drug dx history (n=22) | Orbitofrontal Volume | | | HIV with AUD history (n=10) | Orbitofrontal Volume | | |
|  | rho | p |  |  | rho | p |  |
| Trials 1-5 | .356 | .104 |  | Trials 1-5 | -.042 | .907 |  |
| Free Recall | .354 | .106 |  | Free Recall | -.006 | .987 |  |
| Cued Recall | .380 | .081 |  | Cued Recall | .115 | .751 |  |
| Recognition | .222 | .321 |  | Recognition | .091 | .803 |  |
| Retention: Free Recall | .007 | .974 |  | Retention: Free Recall | -.042 | .907 |  |
| Retention: Cued Recall | -.047 | .836 |  | Retention: Cued Recall | -.273 | .446 |  |
| Note. AUD = Alcohol Use Disorder | | | | | | | |

| Supplementary Table 3:  Spearman Rho Correlations between CVLT scores and Disease-Related Variables in HIV and PD | | | | |
| --- | --- | --- | --- | --- |
|  | Years since Diagnosis | CD4 Nadir | CD4 Current | UPDRS-III |
| **HIV Group** |  |  |  |  |
| Trials 1-5 | .014 | .057 | .100 | -.052 |
| Free Recall | -.088 | -.112 | .034 | .012 |
| Cued Recall | -.084 | .036 | .142 | .051 |
| Recognition | -.038 | -.017 | .262 | .266 |
| Retention: Free Recall | -.016 | -.196 | -.145 | .090 |
| Retention: Cued Recall | .000 | .008 | -.055 | .123 |
| **PD Group** |  |  |  |  |
| Trials 1-5 | -.204 | n/a | n/a | -.148 |
| Free Recall | -.187 | n/a | n/a | -.054 |
| Cued Recall | -.210 | n/a | n/a | -.095 |
| Recognition | -.197 | n/a | n/a | -.049 |
| Retention: Free Recall | -.073 | n/a | n/a | .049 |
| Retention: Cued Recall | -.090 | n/a | n/a | -.031 |
| Bold indicates p<.05 | | | | |
